# Supplementary material for: PanicleNeRF: Low-Cost, High-Precision In-Field Phenotyping of Rice Panicles with Smartphone
Source: Plant Phenomics. 2024 Dec 5;6:0279. doi: 10.34133/plantphenomics.0279 (PMC11617619; doi:10.34133/plantphenomics.0279)
Supplement: Supplementary 1 — Figs. S1 and S2 Tables S1 to S3 Movie S1 [file plantphenomics.0279.f1.zip › Supplementary Information.docx]

Fig. S1. The yellow label used for point cloud scene size calibration in this study. (a) Real-world photograph. (b) Dimensioned schematic.

Fig. S2. Illustration of panicle image annotation for *indica* (upper row) and *japonica* (lower row) rice.

**Table S1.** Model information of key hardware components used for data acquisition and processing in this study

| Hardware component | Model |
| --- | --- |
| Smartphone | iPhone 14 Pro Max |
| Motherboard | MAG X570S |
| GPU | NVIDIA GeForce RTX 4090 (24 GB) |
| CPU | AMD Ryzen 9 5900X |
| RAM | 4 × 32 GB (3600 MT/s) |

**Table S2.** Model information of key hardware components used for web-based platform setup

| Hardware component | Model |
| --- | --- |
| Server architecture | 2U Server (EPYC platform) |
| GPU | NVIDIA GeForce RTX 3090 Turbo Edition (24 GB) |
| CPU | AMD EPYC 7542 |
| RAM | 4 × 64 GB (2933 MT/s) |

**Table S3.** Detailed parameter configurations of models and software utilized in this study

| Model | Parameter |
| --- | --- |
| You Only Look Once version 8 (YOLOv8) | Image size: 640 |
|  | Training set to testing set ratio: 8:2 (480:120) |
|  | Initial learning rate: 0.01 |
|  | Final learning rate: 0.0001 |
|  | Optimizer: SGD |
|  | Batch size: 16 |
|  | Epoch number: 100 |
|  | Parameters size: 3.4 M |
|  | Model file weight: 6.45 MB |
|  | Inference time: 12.1 ms |
| Mask region-based convolutional neural network (Mask-RCNN) | Image size: 640 |
|  | Training set to testing set ratio: 8:2 (480:120) |
|  | Initial learning rate: 0.002 |
|  | Final learning rate: 0.00001 |
|  | Optimizer: SGD |
|  | Batch size: 16 |
|  | Epoch number: 100 |
|  | Parameters size: 63 M |
|  | Model file weight: 483 MB |
|  | Inference time: 72.3 ms |
| Segment Anything Model (SAM) | Model type: ViT-H |
|  | Parameters size: 631 M |
|  | Model file weight: 2.38 GB |
|  | Inference time: 200 ms |
| COLMAP | Version: 3.9.1 |
|  | Max image size: 3200 |
|  | Max number of features: 8192 |
|  | Block size: 50 |
|  | Max number of matches: 32768 |
| Metashape | Version: 2.1.0 |
|  | Accuracy: high (use original image resolution) |
|  | Key point limit: 40,000 |
|  | Tie point limit: 4,000 |
|  | Exclude stationary tie points: true |
|  | Depth filtering: mild |
|  | Calculate point colors: true |
