# Supplementary figures and images for: PanicleNeRF: Low-Cost, High-Precision In-Field Phenotyping of Rice Panicles with Smartphone
Source: Plant Phenomics. 2024 Dec 5;6:0279. doi: 10.34133/plantphenomics.0279 (PMC11617619; doi:10.34133/plantphenomics.0279)

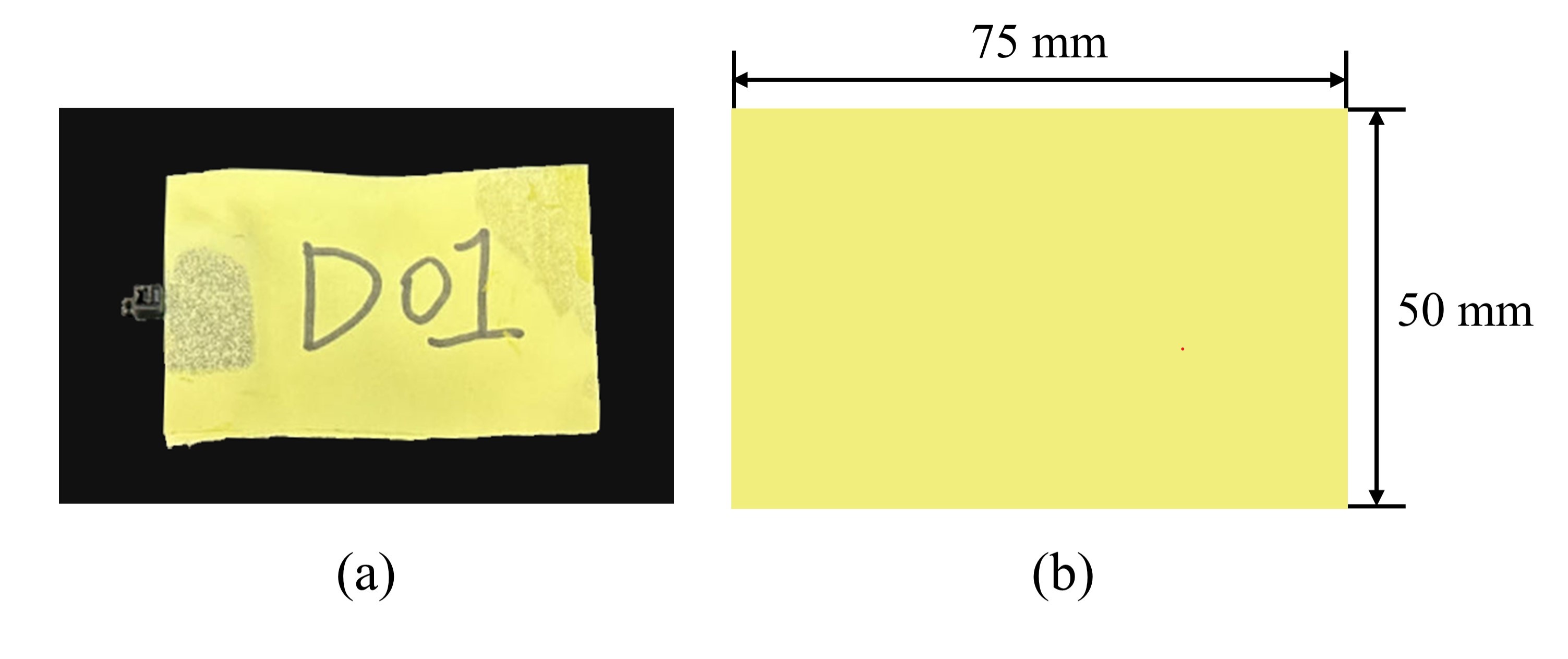

Supplement: Supplementary 1 — Figs. S1 and S2 Tables S1 to S3 Movie S1 [file plantphenomics.0279.f1.zip › Fig. S1 .jpg]

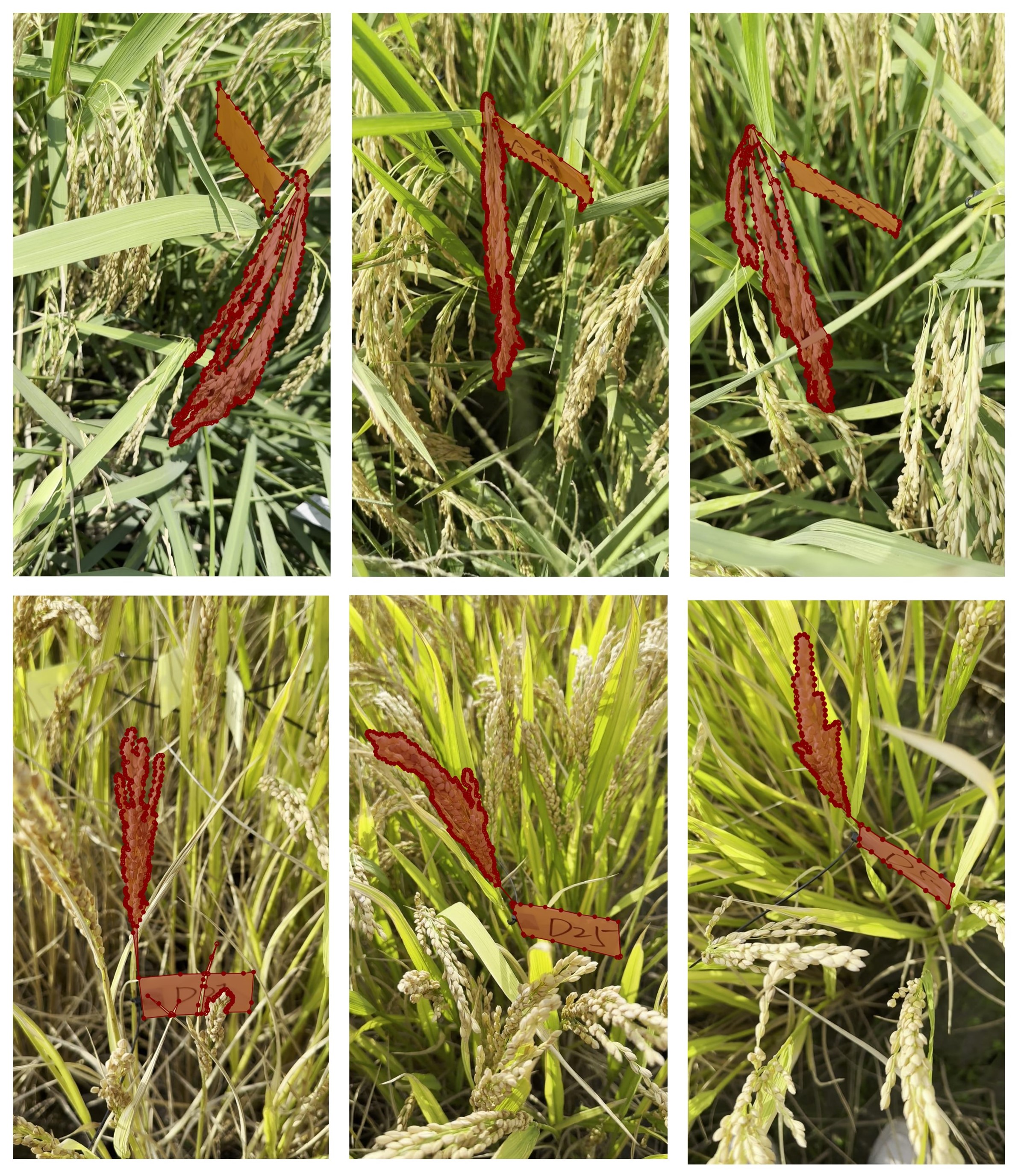

Supplement: Supplementary 1 — Figs. S1 and S2 Tables S1 to S3 Movie S1 [file plantphenomics.0279.f1.zip › Fig. S2 .jpg]
